# A Social Constructionist Influenced Scoping Review of Addictions, Deviance and Crime: Biopsychosocial Perspectives for the Emerging Forensic Mental Health Nursing and Healthcare Services of the Middle East

**DOI:** 10.12688/f1000research.160802.1

**Published:** 2025-01-22

**Authors:** Richard Mottershead, Muhammad Arsyad Subu, Mustafa Habeb, Nafi Alonaizi, Wegdan Bani-Issa, Jacqueline Maria Dias, Fatma Refaat Ahmed, Mini Sara Abraham, Sadeq AL-Fayyadh, Ruwaya Khalfan Saif Almesafri, Ali Alhaiti, Khalid Awad Al-kubaisi, Conrad Murendo, Mohammed Al-Jabri, John Hall, Ghada Shahrour, Chloe Harrison

**Affiliations:** 1 Department of Nursing, College of Health Sciences, University of Sharjah, Sharjah, United Arab Emirates; 2 Military Medical Services, Medical Services, Riyadh, Saudi Arabia; 3 College of Nursing, University of Baghdad, Baghdad, Iraq; 4 Department of Nursing, Faculty of Applied Sciences, Almaarefa University, Riyadh, Saudi Arabia; 5 5. Department of Pharmacy Practice and Pharmacotherapeutics, College of Pharmacy, University of Sharjah, Sharjah, United Arab Emirates; 6 Save the Children, Kabul, Afghanistan; 7 College of Applied Medical Sciences, Nursing Department, Prince Sattam bin Abdulaziz University, Al Kharj, Riyadh Province, Saudi Arabia; 8 SEHA, Sakina, Abu Dhabi Health Services Company, Abu Dhabi, United Arab Emirates; 9 College of Nursing, RAK Medical and Health Sciences University, Ras Al Khaimah, United Arab Emirates; 10 Adferiad, Wales, UK

**Keywords:** Forensic mental health, scoping review, social construction, deviance, addiction, crime, biopsychosocial, Middle East

## Abstract

**Background:**

Nurses and healthcare professionals employed in correctional and forensic mental health settings encounter unique challenges in the care of their patients due to custodial and restrictive environments.

Regions within the Middle East, such as the United Arab Emirates and the Kingdom of Saudi Arabia, have recently experienced exponential economic and healthcare infrastructure development. Mental health has been prioritized for development by recent legislation and practice that incorporate the development of specialist forensic psychiatry services that mediate the need for specialized nurses and allied healthcare staff. Traditionally, forensic care has been provided by general services. The need to progress specialist forensic services with a focus on multidisciplinary staff that seeks to develop safer communities, enhance care, and support the criminal justice system.

**Methods:**

This review article aims to provide a foundation for the nuances of forensic staff through social constructionism. We adopted the framework of Arksey and O’Malley (2005). The use of a scoping review provides a better understanding of the compatibility, content, and outcomes to position the reader to the theoretical construct that society can be seen as existing in both objective and subjective reality.

**Discussion:**

This paper argues for the preparedness of thought understood through social constructionism and demonstrates that it is envisaged that any frequently repeated action becomes cast into a pattern that can be reproduced without much effort. The interconnectedness between the themes of addiction, deviance, and crime allows for a holistic overview and improved understanding for care providers and this was achieved through bio-psychosocial model.

**Conclusion:**

Through the emergence of these complex forms of knowledge, deviance within the lives of patients can be better understood by the emerging professions employed in the emerging forensic healthcare services within the Middle East. These individuals are carefully and dutifully navigating the cultural complexities of mental illness, addictions, and associated deviant behavior.

## Introduction

The relationship between mental health and crime has been prioritized by the WHO (2020) as a necessity to safeguard the mental health of offenders. As a theoretical and conceptual starting point, Crotty’s (1998) research stipulates that the experience of society as a subjective reality is achieved through a primary and secondary process of socialization. By adopting this approach, this study explores addiction and deviance as encountered by patients needing forensic mental health care within the Middle East region. Burr (2003) stipulated that an individual’s identity stems not from inside the person but from the social realm in which they inhabit. In this method, the study will seek to extend the findings of Berger and Luckmann’s (1991) work on socialization in that the processes are enacted upon patients through significant others such as nurses who mediate the objective reality of society, rendering it meaningful; in this way, it is internalized by the individuals (patients). Atkinson (1998) advocates that understanding an individual’s life story can create meaning, which is crucially important for generic healthcare staff who may work in forensic settings within the region. The nation is multicultural, and has a diverse range of languages. Burr (2003) highlighted that language can hold inherent challenges for social constructionism in the accurate transmission of an individual’s thoughts and feelings, but its strength lies in the interpretation of thought in constructing concepts. This paper will demonstrate the importance of developing knowledge about forensic staff, and pertinent issues and concepts that can preempt the development needs of this service within the Middle Eastern region. The authors attempted to provide a means by which knowledge can be explored and realized by nurses and healthcare staff by structuring the way the world is experienced by patients they encounter within forensic settings and how they may then reflect on deviance and addiction. The study extrapolates the ethos of the biopsychosocial model ( Engel, 1977) to explore the phenomena of addictions, substance misuse issues, deviance, and criminality in order to create a holistic understanding. In contrast to the medical model, the bio-psychosocial model of health care ( Engel, 1977) focuses on the patients as ‘experts’ of their own conditions and in the management of their lifestyles ( Carel, 2008). This is particularly important for nurses in forensic settings in the Middle East, who, because of cultural and religious backgrounds, may have little or no understanding of the nuanced lived experiences of patients with criminality and substance misuse needs. Furthermore, it is important to formulate a plan of care for these individuals to support their rehabilitation, recovery, and custodial discharge.

## Methods

### Scoping review

This study undertakes a scoping review of the relevant literature that allows for an exploration of mental health and crime that seeks to support the emergence of forensic mental health as a necessity to safeguard the mental health of offenders and to prioritize the needs of the multidisciplinary teams entrusted to progress forensic mental health in the Middle East.

Arksey and O’Malley (2005) advocate the use of scoping reviews. These authors advocate the use of a scoping review to highlight the need to address broader topics where many different study designs might be applicable and whether there is a challenge in identifying a clear question. Additionally, Arksey and O’Malley (2005) explain that, whereas literature and systematic reviews are predominantly concerned with providing answers to questions from a relatively narrow range of quality-assessed studies, scoping reviews are less inclined to seek to address a specific research question or, consequently, to assess the quality of included studies.

This research strategy of adopting a scoping review, rather than a systematic review, allowed the authors to adopt an evidence-based approach when sourcing relevant studies that allowed for a philosophical exploration with recommendations. Arksey and O’Malley (2005) advocate that an inclusive approach should be adopted. This allowed the authors to review qualitative, quantitative, and randomized control trials within the scoping review. However, it became apparent that there was a challenge in sourcing a breadth of material from the Middle East, so grey literature, theory, or perspectives that may behave relevance to the understanding of the selected topic were applied. Table 1 provides the tabular format for the study’s adherence to this framework.

**Table 1.** Scoping review framework - Six step approach.

1.	Identify the Research Questions
2.	Identify the Relevant Studies
3.	Study Selection
4.	Charting the Data
5.	Collating, Summarising and Reporting
6.	Consult Stakeholders and Policy Makers – Aim: To obtain more references, provide insights on what the literature fails to highlight (Optional).

The co-authors were selected as they are embedded within relevant clinical and educational positions within the region or have specialised professional backgrounds that can add insight into this study’s explorative approach. The authors wished to create an understanding for addictions, deviance, and crime with the inclusion of both historical and contemporary literature. This was formulated as the use of the biopsychosocial model to focus on the findings of the scoping review. This allowed the authors to nurture perspectives and triangulate relevance to the cultural nuances of addiction, crime, and subsequent needs of forensic mental health within the Middle East and North Africa (MENA) region. Therefore, the authors needed to extrapolate and justify a continuation and strengthening of the new paradigm shift to offer evidence for the need to develop forensic healthcare services and strategies within the region.

### Inclusion criteria

Types of sources

This scoping review considered quantitative, qualitative, and mixed-method study designs for inclusion. Documents published from the inception of the database to the present were included. If search parameters were allowed, literature and studies from 1850 to the present were included to allow for the inclusion of seminal literature. Sources were eligible only if the full text was available (e.g., documents available only as abstracts were excluded). Only sources available in English were considered for inclusion because of the limited literature on forensic mental health in the region.

### Study selection

Following the search, all retrieved sources were entered into the reference management software EndNote v.8 (Clarivate Analytics, PA, USA), and duplicate references were removed. Two reviewers independently reviewed the titles and abstracts of all the citations against the inclusion criteria to determine the relevance of the study. If the relevance of a study is unclear in the abstract, then the full article will be reviewed. The full articles of the selected studies will be reviewed independently against the inclusion criteria by two reviewers. The reasons for exclusion of full-text papers that do not meet the inclusion criteria will be recorded and reported in the scoping review. Any disagreements between the reviewers at each stage of the selection process will be resolved through discussion or by a third reviewer.

### Data extraction

This research strategy adopted the scoping review by Arksey and O’Malley (2005). This scoping review model supports an inclusive approach that allows for both sourced literature and insights from the authors. This allowed the authors to review qualitative, quantitative, and randomized control trials within the scoping review and assess their relevance to the cultural and societal needs of the region. This was of benefit, as there was a challenge in sourcing seminal work on forensic mental health within the region, and contextualizing the subject matter with reference to the historical text on addictions, crime, and deviance was the approach adopted to create an understanding of the nursing and healthcare workforce.

### Objective and review question

The author’s rationale for conducting a scoping review was to provide a methodology for determining the state of the evidence on the selected topic, which is especially useful when issues require clarification before further empirical studies can be undertaken. Therefore, the purpose of this study is to adopt Arskey and O’Malley’s scoping review methodology to explore addiction and deviance through a holistic overview of the biopsychosocial approach.

1. What is the extent and nature of published literature that will assist in a biopsychosocial overview of addiction and deviance that will empower a dialogue of the emerging forensic mental health services of the Middle East.

This review paper was conducted to aid in the ongoing dialogue for policy makers to consider when seeking to enhance forensic client-centered frameworks and to examine the training needs of an emerging specialized service focusing on the juxtaposing interplay between mental health and criminology.

## Methodological frameworks: Creating a perspective

Constructionism in this study refers to two forms of understanding: the meaning-making activity of the individual mind, and the collective generation and transmission of meaning in society, as described by Crotty (1998). Crotty’s interpretation was chosen, as he claims that meanings are constructed by human beings as they participate in the world they are interpreting ( Crotty 1998). This approach to social constructionism is based on the studies of Berger and Luckmann (1966), in which all knowledge is socially constructed, including our knowledge of what is real. The landmark contribution to social constructionism by Berger and Luckmann was recognized through their work within The Social Construction of Reality ( Crotty 1998). This epistemological approach was first attached to the work of Immanuel Kant ( Crotty 1998) and this understanding of social constructionism was utilized by notable sociologists of the Chicago Tradition, such as Mead (1934). The authors adopt Crotty’s approach, evident in his earlier work, as it explains that social constructionism creates an awareness that humans (patients) in some respects construct the reality they perceive (1998). This is important to this study, which seeks to create an understanding of the lived experiences of patients within forensic services and how they may interpret their world and, consequently, how their nurses can create meaning about that reality. Therefore, a constructionist approach asserts that concepts are constructed rather than discovered and argues that they correspond to a reality within society ( Hammersley, 1992). Berger and Luckmann (1991) highlight the subtle realism that reality is socially defined but that this reality relates to the subjective experience of individuals’ interactions with ordinary life and how they make meaning of the world rather than to the objective reality of the natural world. This is of crucial importance for the emerging health and social care forensic workforce in the Middle East to explore perceptions of the offending behavior of patients. Articulating this concern, Steedman (2000) believes that the very nature of being human and our attempts to come to an understanding of what this means provide the essence of our need to pursue knowledge, as opposed to scientific inquiry. This paper adopts social constructionism into its exploration, as there is a need for nurses and healthcare staff within the forensic settings within the identified region to understand deviance and addictions and how their patients define their reality and how they perceive it. Therefore, this article is concerned with the nature and construction of knowledge on the issue of addiction, its relationship with deviance, and how this emerging knowledge can have significance for forensic services in the MENA region.

Steedman (2000) emphasized that social constructionism is not concerned with ontological questions or questions of causation. This study argues that both forms of understanding are relevant and necessary for nurses and healthcare staff, as their patients’ experiences can be seen as constructions of an individual nature and constructions that have arisen from engrained political, cultural, and societal influences that have impacted their lives and influenced maladaptive behavior. Consequently, it creates addiction and the presence of deviance and crime as either a causality or a subsequent feature within the lives of patients. Lincoln (2005) argues that constructionism focuses on the fact that humans are social beings who interact with two realities: a physical and temporal reality and an enacted and constructed reality. In reference to forensic patients identified as addicts and deviants, these definitions suggest that their experiences can be understood on at least two levels by their nurses and the multidisciplinary team. Firstly, at the physical and temporal level of reality, the ‘addict’ is an ontological reality that impacts significantly on the patients experience of society ( Klotz, 2004). In relation to Lincoln’s (2005) first reality, Lantman-De Valk, Metsemakers, Haveman, and Crebolder (2000) indicated that an awareness of their lives can be constructed by gaining knowledge of how they are shaped by their substance misuse experiences, which sets them apart or identifies them as different from the rest of a society in which these practices are not accepted or tolerated by the population. Lincoln (2005) explains that these factors (addiction and substance misuse) in a patient’s life may prove challenging to explain solely in a socially, culturally, and politically constructed term. Therefore, Lincoln’s (2005) second reality is relevant to this study as it explores the enacted and constructed level of reality that patients within forensic settings experience and become part of a social categorization system that is based on dichotomies of identity, before their addiction, and importantly for recovery and discharge, after their illicit substance misuse. This is supported by Gallagher (2002), who articulates the point that constructions are socially, culturally, and politically embedded, and can change in different contexts and at a given historical moment. This will be evidenced and explored further within the discussion section on causality and the relationship between addiction, deviance, and crime *.* Crotty (1998) urges caution in that constructionism should be used to reify people (patients) as objects by their offending behavior, as would be the case in the lives of patients. Instead, nurses and healthcare staff may need to consider that reality may exist beyond the way that an individual (addict) makes sense of the ways that they actually are. In creating an awareness of this through Lincoln’s (2005) belief that constructionism can create a focus on the two realities that the patient (addict) within the study provides an insight into these realities. This is an important feature to be explored in the subsequent sections.

Crotty (1998) stipulates that the experience of society as a subjective reality is achieved through a primary and secondary process of socialization. By adopting this approach, this paper explores the identity of addicts and deviants as constructed by society. Burr (2003) stipulated that an individual’s identity stems not from inside the person but from the social realm in which they inhabit. This is important for this study, which examines societal perceptions of the addict engaged in deviance and crime and how understanding can be created through this methodological process for multidisciplinary professions within the Middle East.

In this approach, the study will seek to extend the findings of Berger and Luckmann’s (1991) work on socialization in that the processes are enacted upon the patients through significant others who mediate the objective reality of society, rendering it meaningful and in this way internalized by patients with addictions and substance misuse issues. According to Plummer (2001), this point is stressed in that the observers (nurses and wider healthcare practitioners) provide guidance; it is the patient’s voice that determines the frame of reference for their story and the interactions between the individual and social world. For a culturally diverse multidisciplinary workforce, there is a need to conceptualize the relationship and understanding between criminality, deviance, and addiction through a conceptually understood model to improve their skill set and understanding of forensic care needs within the MENA region.

## Discussion – Biopsychosocial Approach: Understanding Criminality, Deviance and Addictions

### Criminality, Deviant Behaviour & Addictions: Causation

To perpetuate understanding, this paper continues with a deliberation on why individuals appear to be drawn into a criminal act with the presence of addictions and substance misuse as causality. The authors will explore the causes of criminal behavior and relate these to the literature on offending and the relationship with addictions and substance misuse. Within the available literature, the causes of criminal behavior are systematically divided into biological, psychological, cognitive, socio-economic, and political explanations. In addition to these fields, the authors believed that there was a need to integrate criminogenic factors into a holistic overview prior to exploring the relationship between addiction, deviance, and crime from the theory and practice relevant to the care of patients within forensic services.

### Criminality, Deviant Behaviour & Addictions: Biological Causes

Conklin (1995) proposed that offenders differ from non-offenders at a physiological and anatomical level, and therefore attribute crime to individual traits and factors. Indeed, this is relevant to patients with a pre-existing tendency towards criminal activity prior to addiction, and substance misuse becomes a feature within their lives. There are a number of early theories, such as Lombroso’s theory of atavism ( 1911) and Conklin’s (1995) theory of somatotypes, that held that criminals were of a substandard breed, far removed from law-abiding members of the public because of hereditary or genetic defective composition inclusive of a propensity towards addictions and substance misuse behaviors.

There has been some progression from these early theories and an inclination to not just categorize individuals as inherently substandard but rather to look for intraindividual causes of criminal behavior and addictions. Barlow and Durand (2005) reviewed research on family, twin, and adoption studies and suggested that there was a genetic influence on criminal behavior, but that the combination of gene-environmental interaction was more plausible, as genetic factors only influenced crime causation in the presence of certain environmental influences such as addiction and substance misuse. Barlow and Durand (2005) theorized that individuals showing abnormally low levels (under-arousal hypothesis) of cortical arousal might cause individuals to engage in stimulation-seeking behaviors such as gambling or substance misuse in order to reduce perceived boredom. Bird (2007) explains that many individuals who engage in inappropriate behavior, which leads them to contact the judicial system, appear to have a natural inclination towards risk-taking behavior. Barlow and Durand (2005) proposed the fearlessness hypothesis, which holds that individuals diagnosed with personality disorders often have difficulty associating certain cues or signs with impending punishment or danger, thereby preventing them from developing an adequate capacity for impulse control. This can be a feature within the profile of patients being cared for within forensic services with addictions and substance misuse issues.

Furthermore, Conklin (1995) highlighted the fact that within the research offenders appear to have lower levels of monoamine oxidase, which in turn has been linked to extreme impulsivity, sensation seeking, childhood hyperactivity, poor academic performance, and high rates of alcohol and substance misuse, all of which are linked to criminality. Within the female offending population, a study by Conklin (1995) found that the criminal activity was more likely to occur during the four days before and four days during the menstruation cycle. Nurses’ and healthcare practitioners’ awareness of these biological factors will assist in enhancing care provision and support holistic care when examined in collaboration with psychological and cognitive causes.

### Criminality, Deviant Behaviour & Addictions: Psychological and Cognitive Causes

Drake et al. (2010) highlight that psychological explanations have to some extent replaced biological explanations of criminality during and after the 20 ^th^ century. They believe that the current overview of those engaged in deviant behavior associated with substance misuse as being psychologically unbalanced has brought about the present philosophical shift in rehabilitation through the use of talking therapies. Brookman et al. (2010) discuss this progression towards psychological explanations linked to the emergence of the psychoanalytical perspective, that is, that individuals are regarded as antisocial by nature and therefore in need of socialization to avoid further addiction and substance misuse behavior. From a psychoanalytical perspective, Becker (1963) believes that deviance and unlawful behavior, such as the use of illegal substances, can be viewed as a result of faulty or inadequate socialization, which could be solved through psychological treatment rather than direct involvement of the judicial system, which could be viewed as an agent of social control, albeit provided to ensure the immediate safety of society.

In the past, psychological causes of deviance and criminal behavior were believed to be located intra-individually in the form of defective development, low intelligence, and psychopathology and were therefore seen as unrelated to the individual’s environment. Researchers such as Hudson (1996) and Steinberg (2001) noted that the psychological effects of interindividual factors such as themes noted within this study of unemployment, poverty, one-parent family, sexual abuse, child hood abuse and neglect, childhood violence, and dysfunctional family relations were possible causes of criminogenic factors associated with substance issues within life stories. Hernstein and Murray (1994) suggested that this change in the view of the possible reasons for psychological causes of crime has led to the development of mental deficiency theory.

The mental deficiency theory identifies offenders as having a generally lower intelligence quotient and, as a result, they are unable to appreciate the reasons for the existence of the law and the consequences of their actions, or are unable or unwilling to control their actions ( Conklin, 1995). Similarly, this theory has drawn criticism by Cullen (1994), who found that the effects of intelligence on deviance and crime were insignificant and further criticized the mental deficiency theory for completely ignoring white-collar crime. However, Hirschi’s control theory ( Hirschi, 2002) supports the belief that psychological factors cannot be viewed as isolated from interpersonal factors. Hirschi’s control theory supports evidence that suggests that individuals who engage in illicit use of alcohol and drugs lack intimate attachments, aspirations, and moral beliefs that connect law-abiding individuals to a conventional way of life (2002).

Specifically relevant to this research is the suggestion by Conklin (1995) that involvement in addictions and substance misuse can create a social stigma that weakens social bonds. This is highly relevant within the Middle East, where criminal activity, addiction, and substance misuse will have stigma and associated shame for perpetrators. In addition, Hirchi’s control theory ( Conklin, 1995) maintains that maladaptive peer relationships in childhood are linked to later deviance, and this study suggests links to the use of substances leading to criminality.

Researchers have not yet demonstrated conclusive evidence linking personality characteristics to criminal behavior, despite the fact that a great number of studies have initially shown tenuous links to various traits ( Conklin, 1995). Tittle (1985) maintains that various conditions, such as coming from a single parent family, having a diagnosis of a personality disorder, and being susceptible to peer pressure, may result in interpersonal insecurity, which may predispose an individual to deviant and criminal activity. Conklin (1995) explains that there has long been an identified link between criminal behavior and traits such as a low frustration threshold, high levels of aggression, and an inability to delay gratification. Research undertaken by Barlow and Durand (2005) adds that impulsivity, defiance, resentment, absence of feelings of remorse or guilt, indifference to the concerns of others, inability to establish and maintain close interpersonal traits, and inability to learn from experience are the typical traits of psychopathic personality disorder. Furthermore, Barlow and Durand (2005) claimed that many individuals with a personality disorder are at significantly elevated levels of risk for criminal behaviors, such as associations with drug use. These researchers suggest that the difference between individuals diagnosed with personality disorder who become criminals and those who do not is intelligence. Carson et al. (1998) highlight research on differences in the quality of socialization in non-offenders, first offenders, and repeat offenders, finding that repeat offenders are the most poorly socialized. Related to this issue is Rubington and Weinberg’s (2015) proposition that the development of a deviant personality is influenced by the response of others to the alleged deviant act. The authors of this article theorize that depending on the identity of the persons responding, approval and disapproval of the act may either facilitate or inhibit the development of the deviant personality as a function of inclusion in or exclusion from a substance user or group associated with addiction. The authors urge awareness that stigma and shame emanating from a deviant or criminal behavior associated with illegal substances could provide a “family,” with a community based on shared beliefs, experiences, and support socializing albeit that the actions maybe deviant in nature and against the rules imposed by society. This factor means that detecting and diverting individuals with addiction and substance misuse behaviors away from judicial and forensic services could become problematic. One solution could be patients undertaking corrective actions through restorative actions found within peer support that utilizes shared experiences. Campbell and Leaver (2003) state that peer support between individuals can help them be more empowered against the distractions caused by stress and coping with substance abuse. This was similar to a feature within the lead authors’ earlier research on the benefits of peer support with those of a shared identity and life experiences ( Mottershead, 2022; Mottershead & Ghisoni, 2021; Mottershead & Alonaizi, 2021, 2023; Mottershead et al. 2023).

In the 1970s, Yochelson and Samenow (1976) identified criminal thought patterns that were supposedly responsible for criminal behavior, and this research was similar to the work undertaken by Walters and White (1988), who claimed that faulty and irrational thinking characterizes lifestyle criminals. Barlow and Durand (2005) believed that individuals with mental disorders such as substance misuse or addiction could process reward and punishment differently from individuals without this diagnosis in that they are less likely to be deterred from a goal due to the lack of reward or the likelihood of punishment. These researchers believed that by utilizing Gray’s model of brain functioning, it was possible to stipulate that individuals with a mental deficiency could have genetically inherited weak behavior inhibition systems and overactive reward systems ( Barlow and Durand, 2005). This explanation, if assimilated by the emerging forensic workforce, aids in explaining why individuals become engaged in addiction and substance misuse, leading to criminality.

### Criminality, Deviant Behaviour & Addictions: Political and Socio-Economic Causes

In exploring social structures as a cause of criminal behavior, Merton (1968) created his theory of anomie, which suggested that normlessness occurs when social structures prevent individuals from reaching culturally approved goals through institutionalized means; consequently, the individual may resort to violations of the law to reach their goals, which society they consider desirable.

Agnew (1995) developed a general strain theory that holds that there are various sources of strain that cause crime. Agnew proposed the actual or anticipated failure to achieve positively valued goals, actual or anticipated removal of positively valued stimuli, and actual or anticipated presentation of negative stimuli. Therefore, this may be seen as three measures of strain that may cause criminal behavior depending on the magnitude, recency, duration, and clustering of stressful events. Agnew (1995) believed that the impact of strain is influenced by individual adaptability, as well as factors such as temperament, intelligence, interpersonal skills, self-efficacy, association with delinquent peers, and conventional social support. Agnew’s (1995) theory is of specific value to understanding the relatedness of addictions, deviance, and crime as it provides an opportunity to explore the factors underlying addict perpetrator crime. It is hypothesized that the strains of ‘actual or anticipated removal of positively valued stimuli’ (e.g., social standing) and ‘actual or anticipated presentation of negative stimuli’ (e.g., addicts and drug users). This strain experienced by patients in forensic services may precipitate a link between crimes committed by those with addiction and substance misuse needs.

Carson et al. (1998) state that historically, societies that experience periods of extensive unemployment have observed an increase in crime. Conklin (1995) believed that there was a complex connection between unemployment and crime, as some crimes could only occur as a result of opportunities found while in employment, whereas others occur because of being unemployed. The current global downturn may contribute to crimes committed due to addiction and substance misuse and may be an indicator of risk assessments that clinical practitioners may observe within forensic services. This feature mirrors research undertaken by Conklin (1995), who explains that steady employment tends to give people a stake in society in which they do not wish to jeopardize by committing crimes. Conklin (1995) theorized that relative deprivation is an important issue in the causation of crime. As Conklin (1995) states, resentment of poverty is more common among the poor in wealthy nations than among people in poor nations. Therefore, it is the perception of an unfair distribution of wealth rather than a person’s actual level of poverty that can violate the law.

This early work by Glaser (1956) provides further insight in explaining that although an individual may initially violate a law by chance or out of ignorance, the social labelling, devaluing, and stigmatization of the individual may cause the deviant aspects of the individual’s behavior to be overemphasized. Nurses and healthcare workers within the Middle East’s forensic services need to be aware of this overemphasis, as it may cause changes in the patient’s self-concept, leading to feelings of unworthiness and hostility towards others in society. A consequence of the labelling may be the attributing factor that observed continued criminal behavior for the addict/substance misuser, associated with a subculture of labelled individuals, and a limitation of opportunities for success through the perceived short-term misplaced benefits of crime. Based on the work of Werdmolder (1997), it is possible to ascertain that for those patients that healthcare workers in the Middle East may encounter, feelings of rejection may be an indication that they have less ties to conventional institutions and that this marginalization could lead to increasingly inadequate socialization as the label of addicts and substance abusers become engrained within their self-image.

## Conclusion

The study’s research question was reached through the adoption of a scoping review and constructionist epistemology to create an understanding of addictions and substance misusers’ experiences and relationships with deviance and crime. The authors argue that a general theory on the relatedness of deviance, crime, addiction, and substance misuse is problematic, as there is considerable variation in both human behavior and the environment to expect one theory to be applicable in all instances and across multiple cultures. However, the authors also contend that it is indeed possible to construct a general theory on the causation of this interrelatedness for the training of nurses and healthcare practitioners, as different phenomena relating to the relationship may be included within a theoretical commonality, so that what appears to be different causes may be expressions of a common casual relatable theme. This study articulates that the relationship between deviance, crime, and addiction should not be simplified into mere casual observations. Rather, the development of a conceptual framework for the rehabilitation of this offender group with significant health needs should be based on the formulated theory for the betterment of patients receiving care. The societal needs underlying criminal behavior related to addictions must also be fully understood to ensure that they are met in rehabilitation and, ultimately, prevention. The study explored the theoretical causality of deviance and crime as it relates to addictions, taking inspiration from the biopsychosocial model ( Engel, 1977) which considered all the factors that contribute to illness, rather than giving primacy to biological factors alone, which will enhance the healthcare workforce’s ability to understand the patients’ experiences of disrupted well-being, as it relates to addictions. These findings assist in developing a further understanding of challenging subgroups within the region. If patients (offenders) with addiction and substance misuse needs are respected as even partially competent agents of their own lives when they afford adequate access to information and resources, it becomes possible to provide a framework in which they can consider their personal and social reasons for acting unlawfully and assist in finding ways to overcome their forensic healthcare needs and eventually contribute positively to society.

## Authors contribution

RM Conceptualisation, all authors writing, editing.

## Data Availability

No data is associated with this article.
