# Supplementary material for: A Social Constructionist Influenced Scoping Review of Addictions, Deviance and Crime: Biopsychosocial Perspectives for the Emerging Forensic Mental Health Nursing and Healthcare Services of the Middle East
Source: F1000Res. 2025 Jan 22;14:120. [Version 1] doi: 10.12688/f1000research.160802.1 (PMC11836559; doi:10.12688/f1000research.160802.1)
Supplement: Peer review history

# Reviewer report: Reviewer response for version 1

**DOI:** 10.5256/f1000research.176752.r363822

**Published:** 2025-02-28

**Authors:** Suzanne Johnson

**Affiliations:** 1 Anglia Ruskin University, Cambridge, UK

This article takes the reader on a journey to deeper understanding of how addiction (defined as a crime, or form of deviance within the paradigm of social constructionism) may be caused by more than the traditionally cited biological determinants. Via the method of the scoping study a broad range of sources are synthesised to highlight the research evidence which supports the author’s argument that addiction (defined as deviance) is caused by a diverse range of factors, thematically similar to those contained within the biopsychosocial model of health and disease. The authors argue that by considering the varied ‘causes’ of addiction, and perceiving ‘addicts’ as a complex embodiment of these ‘causes’ that health practitioners in forensic settings in the MENA region may be better equipped to provide them with effective care and treatment.

 I believe that this article makes important points, and successfully brings together a number of useful sources of evidence related to addiction, subsequently exposing the benefit of approaching addiction the perspective of the biopsychosocial model. Whilst the authors suggested that they would attempt to focus on the Middle East, there is little within the findings specific to this region. Due to a lack of clear reporting of the methodology it is not possible to assess the methodological rigor and make comment on the quality of the data upon which conclusions are drawn, and thereby confidently concluding that MENA based evidence sources could not be retrieved. Therefore, I have a small number of suggestions regarding how this piece of research could be strengthened.

**Introduction**

 The authors make a strong argument for the need to consider addictions from the viewpoint of social constructionism. They claim that this is particularly important “ *for nurses in forensic settings in the Middle East, who because of cultural and religious backgrounds may have little understanding*…’. The reviewer believes that at this juncture the introduction would benefit from three additions. Firstly the inclusion of an explanation of the current mainstream construction of addiction within MENA society, perhaps using a legal, or clinical definition. Since the authors suggest that behaviour is behaviour until society places a value judgement upon it. I believe this detail would be useful to include. Secondly the reviewer believes the introduction could be strengthened through the inclusion of contemporary data on the number of ‘addicts’ within the forensic system, or the burden of disease they represent in the region to provide context. Further I believe that contemporary demographic data on the nursing workforce could be included to clarify why there may be little understanding of the experiences of their patients, and therefore the need for this research.

**Methods**

 The reviewer considers that the authors chose a suitable method by which to conduct their study, and the detail reporting the expertise of the authors provides assurance that all suitable literature is included in the study. It is appreciated that the authors provided description of the selection process to be conducted, however the reviewer believes this section could be improved significantly if the methodology was reported in full. The reviewer concedes that it was not suggested that this scoping review would be conducted or reported according to PRISMA-ScR Guidelines, although the reviewer believes that either reporting according to these guidelines or providing explanation of why not would have been beneficial. It would be useful to readers if the authors included a list of search terms, initial number of articles retrieved, the inclusion and exclusion criteria used to filter findings, the number of articles rejected, any about which there was disagreement amongst the reviewers, and the final number of articles included. The authors state that data was extracted from each source, it would be very useful if the ‘charted’ data was accessible to the reader. Without this transparency it is difficult for the reviewer to assess the quality of the process by which findings were based. This reviewer considered that insights from ethnographic studies reporting the use of substances socially constructed as illegal in the Middle East may have been included; such as Harthi (2020) or Ghiabi (2021). Of course these sources may have been very reasonably excluded, however, without reporting of inclusion and exclusion criteria this cannot be ascertained.

 This is without doubt a useful synthesis upon which further work on the subject may be based. I look forward to reading further output from these authors on this important topic.

Are the rationale for, and objectives of, the Systematic Review clearly stated?

Yes

Is the statistical analysis and its interpretation appropriate?

Not applicable

If this is a Living Systematic Review, is the ‘living’ method appropriate and is the search schedule clearly defined and justified? (‘Living Systematic Review’ or a variation of this term should be included in the title.)

Not applicable

Are sufficient details of the methods and analysis provided to allow replication by others?

Partly

Are the conclusions drawn adequately supported by the results presented in the review?

Yes

Reviewer Expertise:

Identity, Self Image, Mental Health, Recovery, Social Interventions, Human Animal Relations, Realist Methodology, Scoping Reviews,

I confirm that I have read this submission and believe that I have an appropriate level of expertise to confirm that it is of an acceptable scientific standard.

# Reviewer report: Reviewer response for version 1

**DOI:** 10.5256/f1000research.176752.r361968

**Published:** 2025-02-18

**Authors:** Lynne Alexandra Kennedy

**Affiliations:** 1 Qatar University, Doha, Qatar

Thank you for the opportunity to review this paper, which as the authors claim is conducted on a much neglected area of knowledge and practice, in the Middle East. While substance misuse in the Middle East exists, precise estimates of the prevalence and scale of misuse remains unknown, or underreported. Furthermore, in accordance with Islam , the dominant religion in the region, the use of drugs and alcohol are 'haram' or prohibited,  and therefore rarely discussed. Additionally, such considerations are compounded when exploring the intersection with crime, and deviance. People who deviate, are highly stigmatized. Not surprisingly, understanding about the bio-psycho- social factors leading to or influencing substance misuse, especially amongst those who commit crimes, in the Middle East, is limited; substance misuse services are either limited, or at best fragmented, difficult to access. In some Middle East countries the majority of health workers are migrant workers, who receive their training in other countries, or from educators where health is underpinned by the narrow biomedical model of health, associated with Western medicine. Hence the authors aims and objectives to scope the literature to identify a broader theoretical explanation of substance misuse and deviance, is commendable. They propose a scoping review to achieve this aim. Drawing on existing theory of substance misuse, deviance and crime. To my knowledge, and as claimed by the authors, this subject is understudied and is therefore novel,

 They have undertaken an exploration of the academic literature, from a social constructionist standpoint, to examine how the issue, and interpretations of substance misuse, crime and deviance, are  social and culturally constructed; using seminal literature spanning a broad time period. While this is comprehensive and novel, and especially helpful in highlighting the different assumptions within this literature, the material and perspectives are based predominantly on research conducted in the West.

 Given the aforementioned culturally nuanced context of the Middle East, it would be helpful to see this highlighted as a consideration, and if justified, a limitation of this body of literature.

 The authors claim that their study "articulates that the relationship between deviance, crime, and addiction should not be simplified into mere casual observations. Rather, the development of a conceptual framework for the rehabilitation of this offender group with significant health needs should be based on the formulated theory for the betterment of patients receiving care".

 It would strengthen the paper if a more critical stance to this literature, since much of it is published in seminal works, produced, and therefore socially constructed by researchers and philosophers in the West. 

 To what extent does this theory and body of knowledge apply to practice in the Middle East?

 What are the unique social and cultural elements of substance misuse that arise in the Middle East? Are there regional and country differences in the acknowledgement of, and therefore discussion and social construction of substance misuse in this region?. 

 Whilst not the primary aim of this paper, the authors may have missed an opportunity to highlight the above discourse, acknowledging the implications for reconciling practice with existing theory, and proposing new directions for theory enhancement. 

 If the authors can be persuaded to account for this important social and cultural component, then it would enrich the paper, and provide powerful insight. 

 Finally, I was unsure whether or to what extent the six steps of the scoping review process outlined in Figure 1, were adopted or implemented. For example, it would be interesting to know about step 6, was this undertaken, or is this planned. How was the material from the publications identified by the review, was charted for example. This could be of help and interest to others replicating a scoping review using a social constructionist approach.      

 Again, this may align nicely with the above recommendation. If the authors can clarify this further;  can they clarify the alignment between the structure used here in the paper, and the 6 steps of the scoping review. The amendments suggested above are relatively minor, yet would strengthen the paper considerably.

Are the rationale for, and objectives of, the Systematic Review clearly stated?

Yes

Is the statistical analysis and its interpretation appropriate?

Not applicable

If this is a Living Systematic Review, is the ‘living’ method appropriate and is the search schedule clearly defined and justified? (‘Living Systematic Review’ or a variation of this term should be included in the title.)

Not applicable

Are sufficient details of the methods and analysis provided to allow replication by others?

Partly

Are the conclusions drawn adequately supported by the results presented in the review?

Yes

Reviewer Expertise:

Public Health, Qualitative Research. Health and wellbeing, mental health, Salutogenesis, disordered eating; social and cultural context, lived experiences, bio-psycho-social approach.

I confirm that I have read this submission and believe that I have an appropriate level of expertise to confirm that it is of an acceptable scientific standard.

# Response

**Published:** 2025-02-25

**Authors:** Dr. Richard Mottershead

**Affiliations:** College of Health Sciences, University of Sharjah, Sharjah, Sharjah, United Arab Emirates

On behalf of the research team, I would like to thank Prof. Kennedy for her in-depth and thorough review which has enhanced our article and has created careful consideration for future research. 

 As you have identified we have predominantly used seminal research for western literature and this is we believe a starting point to start to explore the landscape of the development and understanding of concepts around forensic services. 

 Your points in how to potentially strengthen the paper are insightful and we hope to take these suggestions forward in future publications. 

 Again, thank you for your support and time taken to undertake your review. 

 Dr. Richard Mottershead

# Reviewer report: Reviewer response for version 1

**DOI:** 10.5256/f1000research.176752.r361963

**Published:** 2025-02-05

**Authors:** Keith Ford

**Affiliations:** 1 University of York, York, UK

This is a thorough and comprehensive study addressing a very important and pertinent area of mental health.

 The scoping was sufficient and addresses the research question well. The methodology is clearly stated and is replicable for future studies if required and the conclusion draws the together findings nicely.

 Overall an important contribution to the body of knowledge.

Are the rationale for, and objectives of, the Systematic Review clearly stated?

Yes

Is the statistical analysis and its interpretation appropriate?

Yes

If this is a Living Systematic Review, is the ‘living’ method appropriate and is the search schedule clearly defined and justified? (‘Living Systematic Review’ or a variation of this term should be included in the title.)

Yes

Are sufficient details of the methods and analysis provided to allow replication by others?

Yes

Are the conclusions drawn adequately supported by the results presented in the review?

Yes

Reviewer Expertise:

Mental health nursing.

I confirm that I have read this submission and believe that I have an appropriate level of expertise to confirm that it is of an acceptable scientific standard.

# Response

**Published:** 2025-02-17

**Authors:** Dr. Richard Mottershead

**Affiliations:** College of Health Sciences, University of Sharjah, Sharjah, Sharjah, United Arab Emirates

On behalf of the research team, I would like to thank Prof. Ford for his valuable time in undertaking this review. Your guidance has contributed to an article that we hope adds knowledge and insight into an emerging nursing and health specialism in the Middle East.
